# Editorial: Mechanisms, thermodynamics and kinetics of ligand binding revealed from molecular simulations and machine learning

**DOI:** 10.3389/fmolb.2023.1139471

**Published:** 2023-01-17

**Authors:** Yinglong Miao, Chia-En A. Chang, Weiliang Zhu, J. Andrew McCammon

**Affiliations:** ^1^ Center for Computational Biology and Department of Molecular Biosciences, University of Kansas, Lawrence, KS, United States; ^2^ Department of Chemistry, University of California, Riverside, Riverside, CA, United States; ^3^ Drug Discovery and Design Center, Shanghai Institute of Materia Medica, Chinese Academy of Sciences, Shanghai, China; ^4^ Departments of Chemistry & Biochemistry and Pharmacology, University of California San Diego, San Diego, San Diego, CA, United States

**Keywords:** ligand binding, thermodynamics, kinetics, molecular docking, molecular dynamics, brownian dynamics, machine learning, mechanisms

Ligand binding plays an essential role in cellular signaling. Detailed understanding of the mechanisms, structures, thermodynamics and kinetics of ligand binding is central to drug discovery in the pharmaceutical industry and academia (Baron and McCammon, 2013; Peng et al., 2019). Despite this critical importance, such tasks remain challenging in computational chemistry and biophysics. Molecular docking has proven useful in rapid virtual screening of small molecules for drug discovery, although it is often difficult to fully incorporate receptor flexibility into the docking calculations. Recent developments in computing hardware and simulation algorithms have enabled molecular dynamics (MD) simulations to capture dynamic ligand binding and dissociation processes. These simulations can then be analyzed to compute both thermodynamic free energies and kinetic rates of ligand binding (Pang and Zhou, 2017; Tang et al., 2017; Nunes-Alves et al., 2020; Wang et al., 2022). In addition, Brownian dynamics simulations have been very efficient in generating a large number of ligand binding trajectories and estimating the binding kinetic rates (Huber and McCammon, 2019; Muñiz-Chicharro et al., 2022). Finally, emerging machine learning techniques have greatly enhanced molecular simulations and facilitated analysis of the simulation trajectories (Glielmo et al., 2021).

This Research Topic is focused on studies of the pathways, mechanisms, free energies and kinetics of ligand binding to target receptors. We encouraged both method development and application papers. Potential techniques used to address these problems include molecular docking, MD, Brownian dynamics, and machine learning approaches. Systems of interest broadly involve ligand binding to any type of receptors, including proteins, nucleic acids, materials, and so on.

Carloni et al. have reviewed recent major advancements in molecular simulation methodologies for predicting dissociation rate (k_off_), a parameter of fundamental importance in drug design. They further discuss the impact of the potential energy function models on the accuracy of the prediction, and provide a perspective from high-performance computing and machine learning for highly efficient and accurate prediction of the constants. Roussey and Dickson have uncovered important factors of host-guest unbinding through detailed analysis of a large dataset of simulation trajectories. They have found that differences in ion densities as well as guest-ion interactions strongly correlate with differences in the probabilities of reactive paths, and play a significant role in the guest unbinding.

Joshi et al. describe the extension of their clever method using curvilinear coordinate-based sampling to study the thermodynamics of rapamycin associating with the FKBP12 enzyme, the first step in the action of this antiproliferative agent. The method uses a multiple-walker umbrella sampling simulation approach to characterizing the protein–protein interaction energetics along the curvilinear paths, and yields binding free energies and mechanistic details of rapamycin binding with wild-type FKBP12 and modifications of these molecules.

Shinobu et al. have optimized practical protocols for a 2D replica-exchange MD (REMD) method that combines generalized replica exchange with solute tempering and replica-exchange umbrella sampling (gREST/REUS). As demonstrated on ligand binding to three protein kinase systems, the method ensures good random walks in the 2D replica spaces, which are important for enhanced sampling of kinase-inhibitor binding.

Chai et al. have carried out multi-microsecond length MD simulations of STK17B in three different states. They observed the conformational dynamics of its P-loop that could flip into the ADP-binding site upon the inhibitor binding to interact with inhibitors and the protein C-lobe, leading to strengthened communications between the C- and N-lobes. Their simulation results could be useful for designing highly selective inhibitors.

Zhang et al. have carried out MD simulations and Molecular Mechanics/Poisson–Boltzmann Surface Area (MM/PBSA) calculations and revealed stronger binding of rivaroxaban in the Y99C mutant of coagulation factor X than in the Y99A mutant. Their simulations have also shown that ligand binding may not only be a dynamic process but also a dynamic state involving multiple binding poses, which could be important for drug design. Cai et al. have performed MD simulations and absolute binding free energy calculations for exploring the drug resistance mechanism of epidermal growth factor receptor (EGFR), a target protein of many non-small cell lung cancer (NSCLC) drugs. They found the binding affinity of ATP to L858R/T790M mutant is higher than that to the L858R mutant, due to the significant changes of the protein conformation and the van der Waals interactions. Their findings could be valuable for designing new drugs for NSCLC.

Girame et al., Garcia-Borràs and Feixas have applied MD simulations to investigate changes in protonation states of in-pathway residues during protein-ligand binding processes. The authors found that binding of benzamidine to trypsin was infrequent when His57 was positively charged, where His57 was part of the catalytic triad and located more than 10 Å away from the gorge of the substrate binding pocket. Their findings illustrate the importance in properly accounting for protonation states of distal residues when using MD simulations to study ligand binding pathways.

Xue et al. have performed MD simulations on glucocorticoid receptor (GR) complexed with cofactor TIF2 and five different agonists. They have uncovered a communication mechanism between the ligand-binding and cofactor-binding pockets, and identified a pair of important residues (D590 and T739) in the allosteric communication pathway, which could be useful for GR-targeted drug discovery. Shen et al. have examined 130+ RORγ complex structures with different agonists and inverse agonists, identified specific changes in the contact interaction for distinguishing active and inactive conformations, and observed essential modes for separating allosteric binding vs canonical binding and active vs inactive structures. Their simulations and analyses have also revealed some essential contacts to the constitutive activity of RORγ.

Huang et al. have built the most likely 3D structures of alpha/beta hydrolase domain-containing 5 (ABHD5) and the ABHD5-ligand complexes by combining various computational and experimental methods. Their simulations have also identified three residues and some hydrophobic interactions important for protein structure, function and the interactions with ligands and membrane.

Xiao et al. have introduced the Protein Allosteric Sites Server (PASSer2.0), which uses a geometry-based algorithm and automated machine learning to predict allosteric sites. The authors tested a total of 204 proteins from the Allosteric Database (ASD) and ASBench database. The server performed well under multiple indicators. It will provide a valuable tool to facilitate allosteric drug discovery. McKay et al. have developed an essential dynamics ensemble docking (EDED) approach to identify the most relevant receptor conformations for virtual screening. They have demonstrated the approach on docking of small-molecule antagonists of the PAC1 class B GPCR. With four representative receptor models selected from simulations and screening of three million ZINC compounds and 23 experimentally validated ligands of PAC1, they show that EDED can effectively reduce the number of false positives and improve the accuracy of docking.

The paper “Big Data Analytics for Improved Prediction of Ligand Binding and Conformational Selection” by Gupta et al. continues the work by these authors to enhance our understanding of the binding of small molecules to proteins through the conformational selection mechanism. The authors make use of modern machine learning approaches and provide valuable tools for identifying proteins that utilize these mechanisms.

In summary, remarkable advances have been made in both method development and applications in computational predictions of ligand binding free energies and kinetics (especially the dissociation rate). Advanced MD simulations have revealed mechanisms of ligand recognition and associated protein conformational changes, which often involves allosteric modulation. Novel approaches have been developed to select important receptor conformations for molecular docking and improve the docking accuracy. A new server (PASSer2.0) has been developed for predicting allosteric sites in proteins based on machine learning. It will greatly facilitate allosteric drug discovery. These advances are expected to expand our capabilities in simulations of ligand binding and drug discovery.
